# Identification of 2-Amino Benzothiazoles with Bactericidal Activity against Mycobacterium tuberculosis

**DOI:** 10.1128/spectrum.04974-22

**Published:** 2023-01-23

**Authors:** Shilah Bonnett, Jo-Ann Jee, Somsundaram Chettiar, Yulia Ovechkina, Aaron Korkegian, Eric Greve, Joshua Odingo, Tanya Parish

**Affiliations:** a TB Discovery Research, Infectious Disease Research Institute, Seattle, Washington, USA; b Center for Global Infectious Disease Research, Seattle Children's Research Institute, Seattle, Washington, USA; Johns Hopkins University School of Medicine

**Keywords:** antitubercular, drug discovery, antibiotic, protein secretion, bactericidal activity, tuberculosis

## Abstract

We identified an amino-benzothiazole scaffold from a whole-cell screen against recombinant Mycobacterium tuberculosis under expressing the essential signal peptidase LepB. The seed molecule had 2-fold higher activity against the LepB hypomorph. Through a combination of purchase and chemical synthesis, we explored the structure-activity relationship for this series; 34 analogs were tested for antitubercular activity and for cytotoxicity against eukaryotic cells. We identified molecules with improved potency and reduced cytotoxicity. However, molecules did not appear to target LepB directly and did not inhibit protein secretion. Key compounds showed good permeability, low protein binding, and lack of CYP inhibition, but metabolic stability was poor with short half-lives. The seed molecule showed good bactericidal activity against both replicating and nonreplicating bacteria, as well as potency against intracellular M. tuberculosis in murine macrophages. Overall, the microbiological properties of the series are attractive if metabolic stability can be improved, and identification of the target could assist in the development of this series.

**IMPORTANCE** Mycobacterium tuberculosis, the causative agent of tuberculosis, is a serious global health problem requiring the development of new therapeutics. We previously ran a high-throughput screen and identified a series of compounds with antitubercular activity. In this paper, we test analogs of our hit molecules for activity against M. tuberculosis, as well as for activity against eukaryotic cells. We identified molecules with improved selectivity. Our molecules killed both replicating and nonreplicating bacteria but did not work by targeting protein secretion.

## INTRODUCTION

Tuberculosis remains a major public health threat with more than 1 million people dying annually and approximately millions of new cases per year (1). The COVID-19 global pandemic has considerably worsened the situation and may lead to increased cases of tuberculosis due to the lack of diagnosis and treatment. Although there has been a large increase in the number of studies focused on identifying new tuberculosis drugs, the drug pipeline remains weak due to high attrition rates and the lack of new classes of drugs.

We are interested in identifying and progressing new molecular scaffolds with potential as antitubercular agents. Phenotypic screening using high-throughput assays to identify agents from large molecular libraries has proved to be a good starting point with multiple new classes at the earliest stages of discovery (2–7). A number of screening approaches have been adopted including aerobic replicating growth in standard medium, various carbon sources, nutrient and/or oxygen starvation, acidic conditions, and combinations of all these factors in multistress models, as well as intracellular growth in macrophages and in multicellular granuloma models (5, 7–15).

Biochemical screening to identify compounds with activity against M. tuberculosis has been largely unsuccessful due to their lack of whole-cell activity (16). Alternative approaches to using hypomorph strains have been developed, which enable the identification of additional chemical scaffolds (which may have weak activity against a wild-type strain) (17, 18). The advantage of such an approach can be that target identification is more rapid. We are interested in the Sec pathway of protein secretion. Secretion via the Sec pathway is essential for M. tuberculosis viability and we previously confirmed that LepB itself is essential under standard growth conditions (17). We have previously developed and run a screen against a strain of M. tuberculosis that underexpresses the essential signal peptidase LepB in an attempt to identify inhibitors of protein secretion via the Sec pathway (17). We report the exploration of a hit scaffold from this screen in this paper.

## RESULTS AND DISCUSSION

### Identification of novel inhibitors of M. tuberculosis growth.

**FIG 1:**
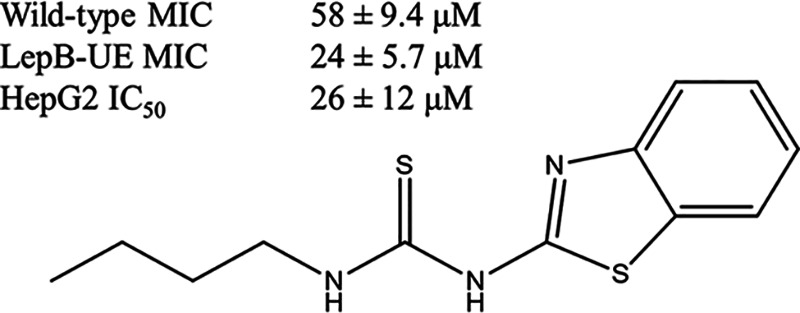
The seed benzothiazole molecule (molecule 1). The molecule was identified in a screen against M. tuberculosis underexpressing the sole signal peptidase. MICs were determined against this hypomorphic strain (LepB-UE) and the wild-type strain. Cytotoxicity was measured against HepG2 cells. Data are the average ± standard deviation (*n* = 4 for MICs).

We previously ran a high-throughput screen against two strains of M. tuberculosis to find novel inhibitors of mycobacterial growth (17, 19). Using a strain of M. tuberculosis that had lower expression of the signal peptidase LepB, we identified a single benzothiazole with modest antitubercular activity (Fig. 1).

**FIG 2:**
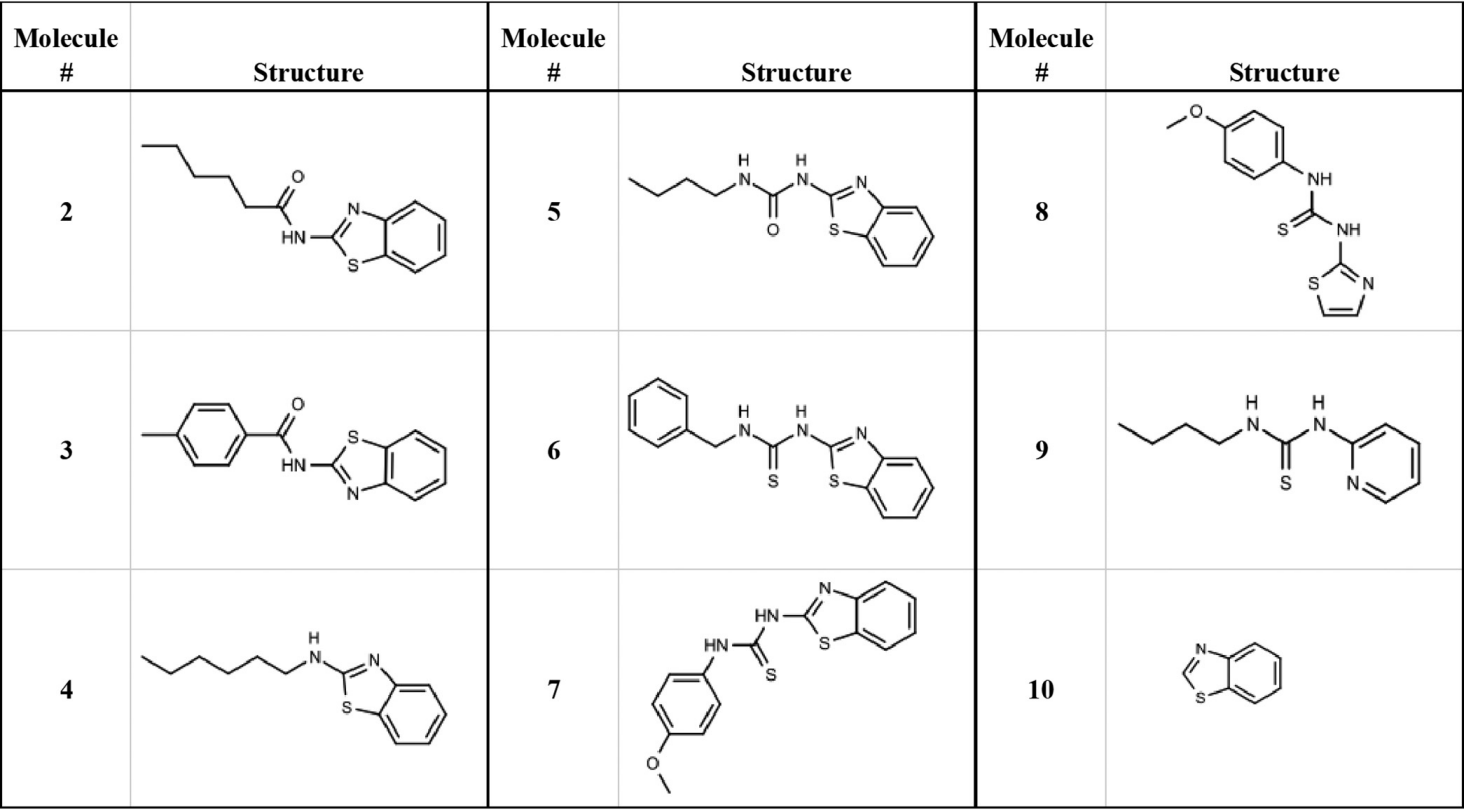
Molecules purchased for hit evaluation.

This molecule had ~2-fold higher activity against the LepB-UE strain (*P* < 0.005). To determine if similar molecules had activity, we purchased a number of analogs and tested these for activity against the wild-type and LepB-UE strains (Fig. 2 and Table 1). Only two additional molecules (4 and 8) had activity against the wild-type strain with modest MICs of 125 μM and 27 μM, respectively. However, six of the molecules had reasonable activity against the LepB-UE, with five having MIC <100 μM ranging from 14 to 82 μM (molecules 1, 4, 5, 6, and 8). We also measured cytotoxicity against the human HepG2 cell line. Nine of the molecules were cytotoxic, but the activity did not track with antitubercular activity suggesting that the two biological activities could be separated. Based on these data, we decided to conduct a hit assessment to determine the tractability of the series. We designed and synthesized a small set of analogs to explore the structure-activity relationship with a focus on improving potency against the wild-type strain and eliminating cytotoxicity.

**TABLE 1:** Activity of commercially available analogs^*a*^

Molecule no.	MIC (μM)		IC_50_ (μM)
	Wild type	LepB-UE	HepG2
1	47 ± 12	25 ± 4.5	26 ± 12
2	>200	>200	74 ± 13
3	>200	140^*b*^	>100
4	125 ± 13	82 ± 16	55 ± 8.5
5	>200	61 ± 9.1	39 ± 25
6	>200	14^*a*^	6.0 ± 0.1
7	>200	>200	>100
8	27 ± 17	18 ± 2.0	21 ± 19
9	>200	100^*a*^	65 ± 50
10	>200	>100^*a*^	7.6 ± 6.2

a Data are average and standard deviation of a minimum of 2 biological replicates except where indicated. MIC, concentration required to inhibit growth by 90%; IC_50_, concentration required to inhibit growth by 50%.

b *n* = 1 replicate.

### Structure-activity relationship for the benzothiazole series.

We first explored the benzothiazole (BET) core. In our first iteration, we synthesized three new molecules; all were tested for activity against the wild-type and LepB-UE strains, as well as for cytotoxicity for HepG2 cells (Table 2). Replacing the benzothiazole with a benzimidazole (molecule 11) or benzoxazole (molecule 12) retained good potency against the LepB-UE strain, while the benzoxazole led to improved activity against the wild-type strain that was equivalent to the LepB-UE strain (MIC of 23 to 32 μM), but both molecules were cytotoxic. Adding a chloro-substituent (molecule 13) at position 4 increased activity against the LepB-UE strain (MIC = 7.9 μM) and reduced cytotoxicity (HepG2 IC_50_ >100 μM) but did not improve potency against the wild-type strain.

**TABLE 2:**
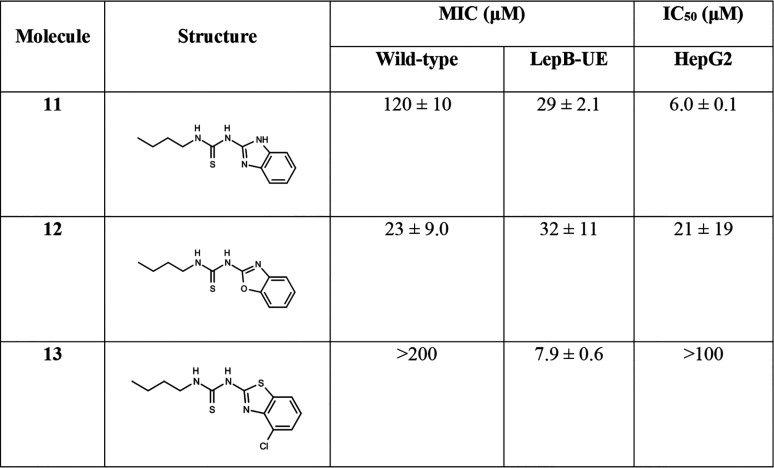
Activity of core analogs^*a*^

a Data are average and standard deviation of a minimum of 2 biological replicates. MIC, concentration required to inhibit growth by 90%; IC_50_, concentration required to inhibit growth by 50%.

Second, we simplified the benzothiazole to a single-ring system. We synthesized 10 analogs and tested them for biological activity (Table 3).

**TABLE 3:**
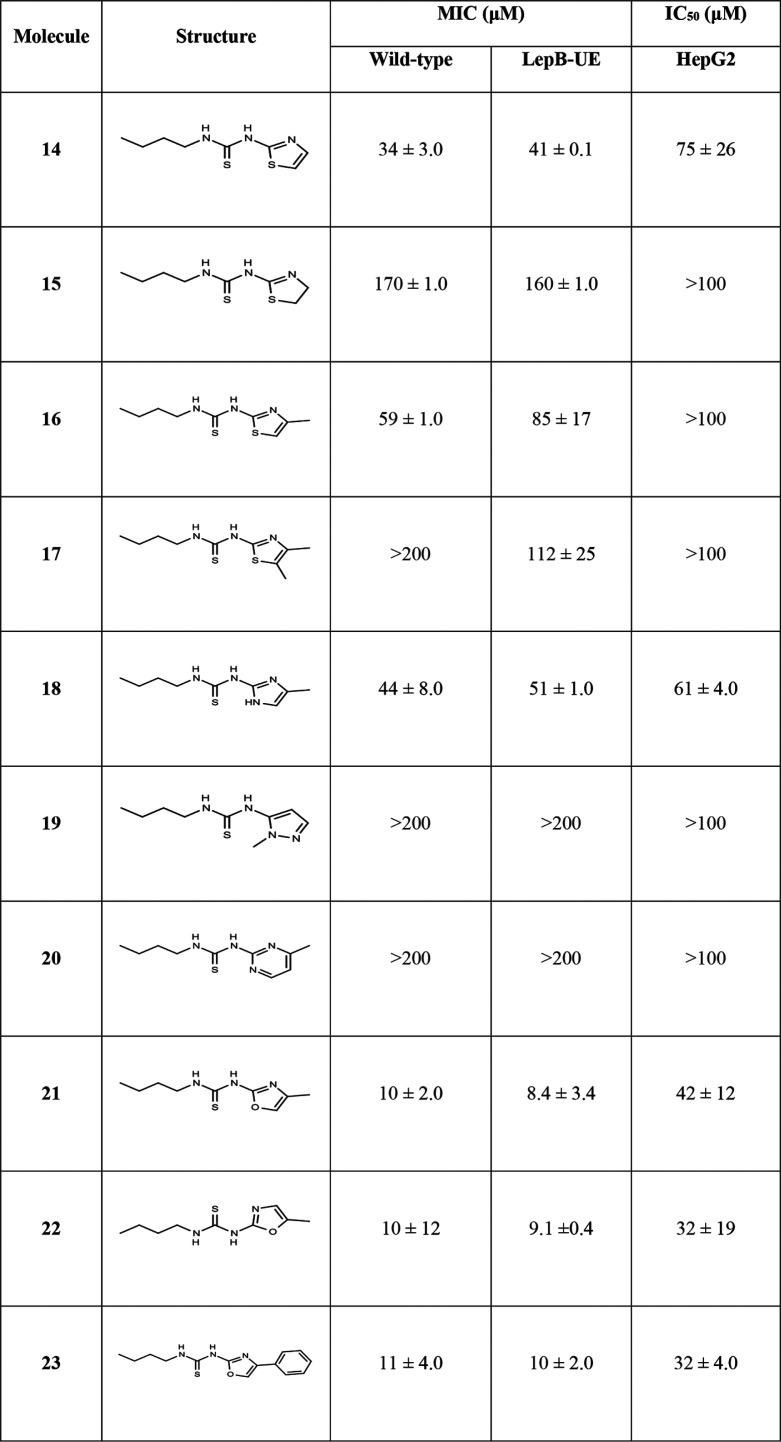
Activity of oxazole and thiazole core analogs^*a*^

a Data are average and standard deviation of a minimum of 2 biological replicates. MIC, concentration required to inhibit growth by 90%; IC_50_, concentration required to inhibit growth by 50%.

Compared to compound 1, compound 14 with the 1,3 thiazole had improved potency against the wild-type strain (34 μM compared to 120 μM) but no increased activity against the LepB-UE strain (MICs = 29 μM and 41 μM). Removal of a double bond to give compound 15 resulted in a 4- to 5-fold loss of activity with no differential activity (MIC = 170 μM and 160 μM against wild-type and LepB-UE strains, respectively). Adding a methyl group at position 5 (compound 16) or 4,5 dimethyl group (compound 17) did not improve activity. Overall, the thiazole derivatives were less cytotoxic than their benzothiazoles (HepG2 IC_50_ >100 μM), with only compound 15 demonstrating any cytotoxicity (HepG2 IC_50_ = 75 μM). Replacing the sulfur (compound 17) with nitrogen (compound 18) retained activity against both strains (MIC = 44 μM and 51 μM for wild-type and LepB-UE, respectively), but this molecule was cytotoxic (HepG2 IC_50_ = 61 μM). Replacing the thiazole with a methylated pyrazole (compound 19) or methylated pyrimidine (compound 20) resulted in the loss of activity against both strains, but these molecules were not cytotoxic. Methylation of the oxazole at either position 4 or 5 (compounds 21 and 22) greatly improved activity against both strains (MICs = 10 μM against the wild type; MICs = 8 to 9 μM LepB UE strains). Although both molecules had some cytotoxicity (HepG2 IC_50_ = 42 and 32 μM for compounds 21 and 22, respectively), there was improved selectivity over earlier analogs. The addition of a phenyl group (compound 23) had a similar effect with improved activity and some cytotoxicity (MICs = 10 to 11 μM; HepG2 IC_50_ = 32 μM).

Next, we explored the left-hand side of the molecule (Table 4). The inclusion of an oxygen or sulfur in the alkyl chain (compounds 24 and 25) resulted in some activity against the wild type but resulted in increased cytotoxicity and loss of selectivity. The addition of a phenyl group (compound 26) increased activity against the LepB-UE strain, but no activity against the wild-type strain. The addition of a methyl group on the thiourea (compound 27) greatly increased cytotoxicity (HepG2 IC_50_ = 5.6 μM) without improving antitubercular activity (MIC = 155 to 195 μM). Replacing the alkyl chain with an isopropyl group resulted in good activity for both the benzothiazole (compound 28) and oxazole derivatives (compound 29), but no selectivity (MICs = 20 to 40 μM against wild type; MICs = 12 to 21 μM against LepB-UE; HepG2 IC_50_ = 27 to 78 μM).

**TABLE 4:**
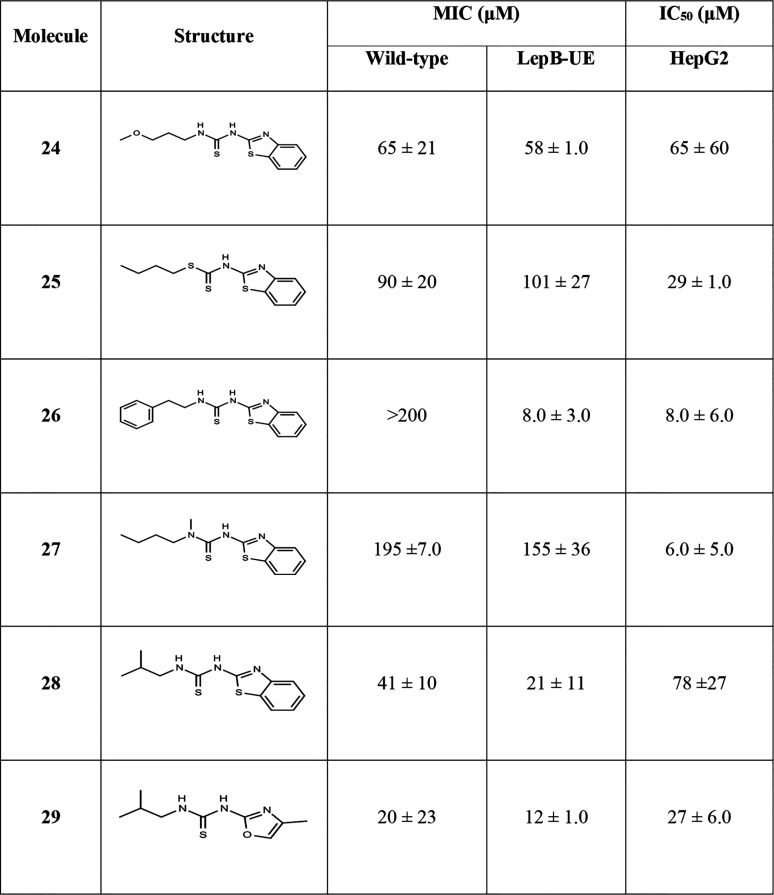
Activity of side chain core analogs^*a*^

a Data are average and standard deviation of a minimum of 2 biological replicates. MIC, concentration required to inhibit growth by 90%; IC_50_, concentration required to inhibit growth by 50%.

Finally, we made further changes to the molecule on both the left- and right-hand sides (Table 5). Switching the thiourea to the benzene ring from the thiazole (compound 30) reduced cytotoxicity but lost ~2-fold biological activity. Since we had seen an improvement in selectivity by adding a chloro-substituent in molecule 13, we made another analog with chloro at the 4 position (compound 31); this also abrogated cytotoxicity (HepG2 IC_50_ >100 μM), while retaining activity against the LepB-UE strain (MIC = 14 μM) but no activity against the wild-type strain. Since the oxazoles were generally more active, we synthesized benzoxazoles with chloro-substituents on the 5, 6, and 7 positions (compounds 32–34); however, this did not improve selectivity and reduced biological activity. Finally, we added a methoxy group to the benzoxazole (compound 35), but this abrogated all activity, although the molecule was not toxic.

**TABLE 5:**
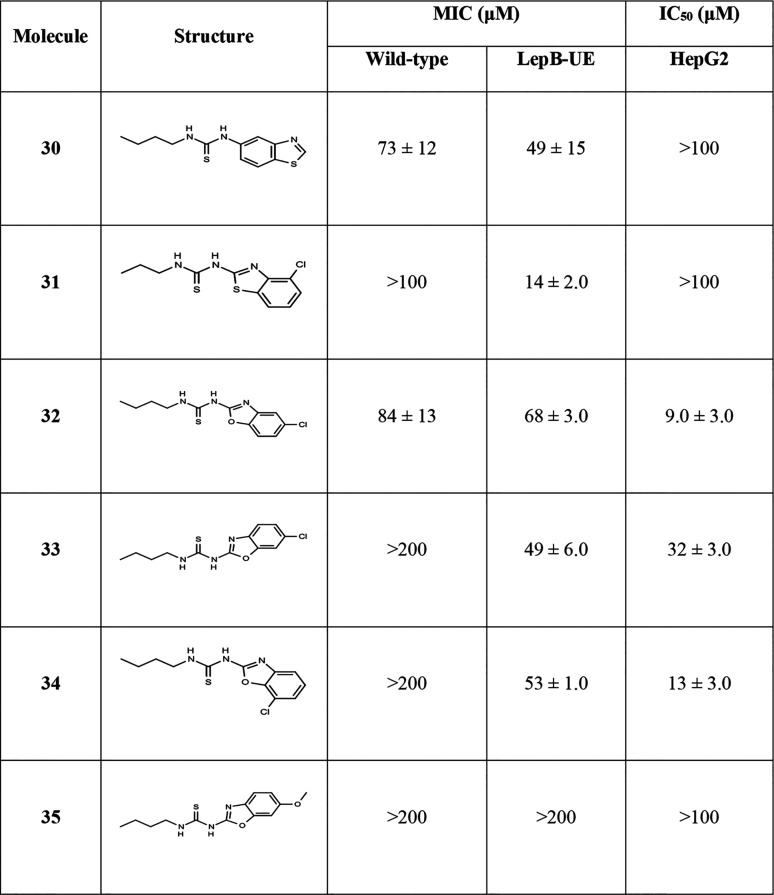
Activity of analogs^*a*^

a Data are average and standard deviation of a minimum of 2 biological replicates. MIC, concentration required to inhibit growth by 90%; IC_50_, concentration required to inhibit growth by 50%.

### BET molecules do not inhibit protein secretion.

**FIG 3:**
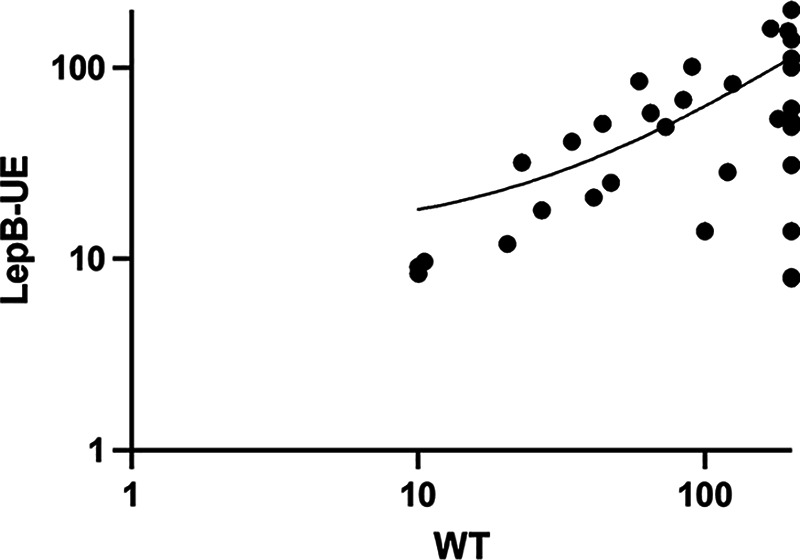
Correlation between activity against wild-type (WT) and LepB-UE strains. The average MICs for each strain are plotted. The Pearson correlation coefficient *R*^2^ = 0.32. LepB-UE, M. tuberculosis strain under expressing LepB; WT, M. tuberculosis wild-type strain.

We originally identified the seed molecule for this series from a screen against the LepB-UE strain and confirmed that it had differential activity, i.e., it was more effective against the LepB-UE strain (Fig. 1). However, our structure-activity relationship studies did not see any increased activity against the LepB-UE strain (Fig. 3). To determine if molecules target the secretory pathway, we tested a subset for inhibition of protein secretion via the Sec pathway in membrane fractions of M. tuberculosis using a fluorogenic peptide substrate (Table S1 in the supplemental material). Fourteen molecules representative of the series were tested, but none showed inhibition of secretion up to 200 μM, suggesting that they work via another mechanism.

### BET molecules are active against intracellular M. tuberculosis.

We tested a small subset of molecules for activity against M. tuberculosis cultured in murine macrophage using a high-content microscopy method. This enabled us to test cytotoxicity against infected macrophages simultaneously to reduce artifacts, since toxicity to the macrophages would also prevent mycobacterial growth (Table 6). Three molecules had activity against intracellular M. tuberculosis, and two of these molecules (1 and 23) had excellent potency of <10 μM. Interestingly, the activity against intracellular bacteria was far greater than that against aerobically cultured organisms. We were unable to measure intracellular activity for the remaining six molecules tested due to cytotoxicity against the murine macrophages. Activity against intracellular organisms is of particular interest since there is increasing evidence to link intracellular bacteria with both increased heterogeneity and antibiotic tolerance (20, 21); thus, targeting this population could be useful in a new regimen.

**TABLE 6:** Activity against intracellular M. tuberculosis^*a*^

Molecule no.	IC_50_ (μM)	
	MTB	Macrophage
1	2.5 ± 0.4	>100
13	NC	12 ± 2.8
21	NC	6.0 ± 1.0
22	NC	7.1 ± 1.5
23	1.3 ± 0.4	30 ± 6.4
26	NC	1.4 ± 0.2
29	NC	8.1 ± 1.6
31	11 ± 2.2	>100
34	NC	5.8 ± 0.4

a Data are average and standard deviation of a minimum of 2 biological replicates. IC_50_, concentration required to inhibit growth by 50% for either intracellular M. tuberculosis (MTB) or the RAW264:7 cells (macrophage); NC, not calculated due to cytotoxicity against RAW264:7 cells.

### BET molecules have bactericidal activity against M. tuberculosis.

**FIG 4:**
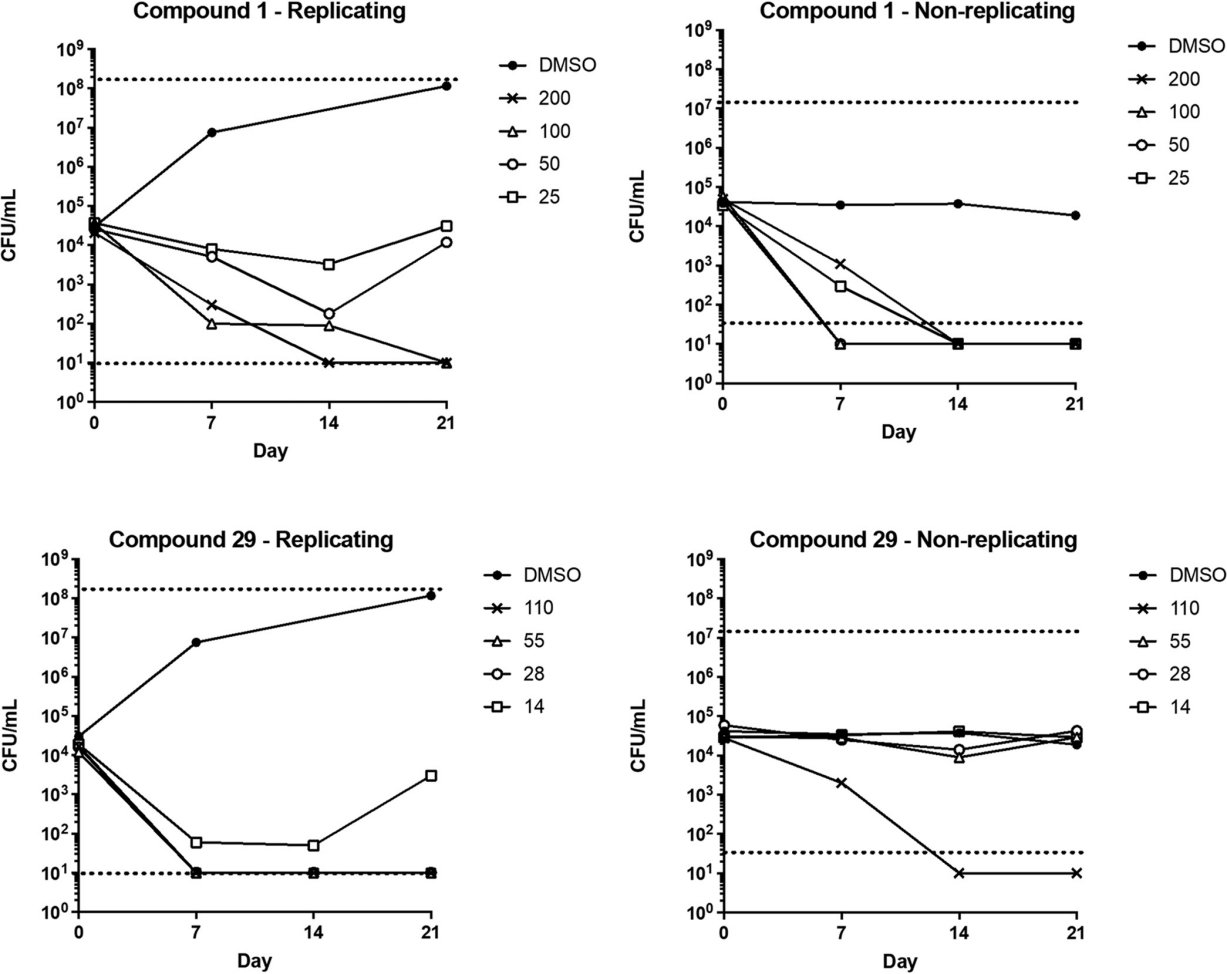
Kill kinetics for Compounds 1 and 29 against M. tuberculosis. Replicating bacteria were cultured in standard medium, nonreplicating bacteria were in PBS-tyloxapol. Compounds were added at the indicated concentrations (μM). Viable bacteria were measured as CFU.

We tested two molecules for their ability to kill M. tuberculosis under replicating (aerobic growth) and nonreplicating (nutrient starvation) conditions (Fig. 4). Both molecules were able to kill replicating organisms with complete sterilization of replicating cultures at 100 μM after 21 days. Compound 29 was more effective with bactericidal activity (defined as 3 log kill) at 27.5 μM. In contrast, compound 1 was more effective at killing nonreplicating organisms with sterilization at the lowest concentration tested (20 μM), whereas compound 29 was only effective at the highest concentration (100 μM). This may indicate that the molecules have a different mode of action.

### ADME properties.

We selected five compounds including the original seed compound for *in vitro* absorption, distribution, metabolism, and excretion (ADME) studies. We prioritized compounds with the lowest cytotoxicity, while selecting compounds with different structural features. Three molecules showed good permeability determined using Caco-2 cells, and none of the five were subject to efflux (Table 7 and Table S2). Microsomal stability was generally low with a half-life in the presence of human microsomes ranging from 8 to 26 min (Table 8 and Table S3). The molecules were generally clean in cytochrome P540 inhibition assays (Table S4), except that three molecules inhibited CYP2C19. Protein binding was variable ranging from 94.5% for compound 26 to 99.9% for compound 1 (Table S5). These data suggest that the key liability for the series is metabolic stability.

**TABLE 7:** Permeability of molecules^*a*^

Molecule no.	Papp (10^−6^ cm s^−1^)		
	Mean a→B papp (10^−6^ cm/s)	Mean B→a papp (10^−6^ cm/s)	Efflux ratio (re) ratio
1	1.4	2.5	1.7
12	4.9	5.8	1.2
14	22	18	0.81
21	21	19	0.89
24	17	15	0.87
Atenolol	0.13	0.3	2.4
Propanolol	20	30	1.5
Talinolol	0.033	5.3	161

a Data are expressed as permeability (10**^−^**^6 ^cm s**^−^**^1^). The permeability coefficient (Papp) was measured using Caco-2 cells. (A→B), apical to basolateral; (B→A), basolateral to apical; Efflux ratio (Re), ratio calculated as Papp(B→A)/Papp(A→B); atenolol, low permeability control; propanolol, high permeability control; talinolol, P-glycoprotein efflux control; Papp, (dQ/d*t*)**/**C0 × *A*; dQ/d*t*, rate of permeation; C0, initial concentration of test agent; *A*, area of monolayer.

**TABLE 8:** Metabolic stability^*a*^

Molecule no.	CL_int_ (μL min^−1^ mg^−1^)	*t*_1/2_ (min)
1	96	24
12	218	11
14	89	26
21	179	13
24	282	8.2
Verapamil	128	18
Dextromethorphan	19.5	118

a Data are average of two replicates. NADPH-dependent clearance (CL_int_) and half-life (*t*_1/2_) were measured using human liver microsomes with test compounds at 1 μM. Verapamil and dextromethorphan were used as controls for high and low clearance, respectively. CL_int_ = ln(2)**/**(*t*_1/2_ [microsomal protein]).

To our knowledge, this is the first report of the activity and SAR against M. tuberculosis for benzothiazoles and benzoxazoles containing a urea or thiourea substituent, although similar molecules have been reported as active for different activities such as myorelaxants (22). Previous work has identified benzothiazole amide derivatives as active against both M. tuberculosis and nontuberculous mycobacteria (22, 23), and a single benzothiazole had weak activity against M. tuberculosis (24), but no urea derivatives were included, and the majority of the compounds were substituted on the cyclohexane ring. The related antitubercular thioalkyl benzoxazole is proposed to be metabolized by M. tuberculosis MymA to the active form (25). EthA and MymA are mono-oxygenases that can activate sulfur-containing prodrugs, and it is possible that the molecules we describe are activated by the same mechanism (26). However, it is not clear why strains with lowered levels of the signal peptidase LepB would be more sensitive to prodrugs, and neither EthA nor MymA is a secreted protein. We determined that the molecules in this series did not work by inhibiting secretion in M. tuberculosis. There are other possibilities for the differential activity between the wild-type and LepB-UE strain. In particular, there could be changes in the cell wall, which alter either molecule permeation and/or efflux thereby affecting the intracellular concentration.

We explored a series of molecules based around a benzothiazole seed molecule for their potential as new antitubercular agents. We conducted preliminary structure-activity relationships and identified molecules with improved potency and reduced cytotoxicity, as well as molecules with excellent intracellular activity and selectivity. The key liability for the series is metabolic stability. The series does not appear to target protein secretion and further work to investigate the mode of action could assist in the development of this series by allowing for a structure-based design.

## MATERIALS AND METHODS

### MIC and kill kinetics.

M. tuberculosis was cultured in Middlebrook 7H9 medium supplements with 10% vol/vol OADC supplement (Becton Dickinson) and 0.05% wt/vol Tween 80. CFU were determined on Middlebrook 7H10 agar with 10% vol/vol OADC after 3 to 4 weeks incubation. MICs against M. tuberculosis were determined as described previously (27). Growth was measured by optical density at 590 nm and relative fluorescence intensity after 5 days culture. MIC was defined as the concentration of compound required to inhibit growth of M. tuberculosis by 90% and was determined using the Levenberg-Marquardt least-squares plot. Rifampicin controls (maximum inhibition) and a rifampicin dose response were included on every plate. Bacterial viability was measured in log phase cells or in cells previously starved for >4 days in PBS, 0.05% wt/vol tyloxapol (28). Compounds were added at multiples of the MIC (at the time the assay was initiated).

### HepG2 cytotoxicity.

HepG2 cells were used to measure eukaryotic cytotoxicity. Viability of HepG2 was measured using CellTiter-Glo reagent (Promega) after 3 days of exposure to compounds. IC_50_ was calculated as the compound concentration required to reduce cell viability by 50% and was determined using the Levenberg-Marquardt least-squares plot. Staurosporine controls (maximum inhibition) and a staurosporine dose response were included on every plate.

### Activity against intracellular M. tuberculosis.

Murine RAW 264.7 macrophages (ATCC TIB-71) were infected with M. tuberculosis DsRed (DREAM8) (29) as described at a multiplicity of infection of 1 for 24 h, washed, harvested, and dispensed into 384-well plates (30). Infected macrophages were exposed to compounds for 72 h; cell nuclei were stained with SYBR green I. Plates were imaged with an ImageXpress Micro High Content Screening System (Molecular Devices) using a ×4 objective and FITC and Texas Red channels. MetaXpress was used to determine the integrated intensity of bacteria or macrophages. IC_50_ was calculated as the compound concentration required to reduce bacterial growth by 50%. TC_50_ was calculated as the compound concentration required to reduce macrophage viability by 50%. Isoniazid and staurosporine controls (maximum inhibition) and dose responses were included on every plate.

### ADME.

Microsomal stability was measured over 45 min (0-, 5-, 15-, 30-, and 45-min samples) with 1 μM compound, 0.3 mg/mL human microsomes in 100 mM potassium phosphate buffer pH 7.4, and 3 mM MgCl_2_ plus 2 mM NADPH where indicated. Intrinsic clearance was determined as ln(2)/(*t*_1/2_ [microsomal protein]), where *t*_1/2_ is the half-life. Permeability was measured across Caco-2 cells cultured in a Millipore 96-well Caco-2 plate. For apical to basolateral (A→B) permeability, 10 μM compound was added to the apical (A) side and amount of permeation was determined on the basolateral (B) side; for basolateral to apical (B→A) permeability, the compound was added to the B side and the amount of permeation was determine on the A side. Time points were taken at 2 h.

### General synthetic procedures.

Unless otherwise noted, all chemicals used were purchased pure, from commercially available sources such as Sigma-Aldrich, VWR, Fisher or other chemical vendors. All reagents and solvents were used as received. ^1^H nuclear magnetic resonance (NMR) (300 MHz) spectra were recorded on a Bruker Biospin NMR spectrometer. Peak multiplicities are denoted as follows: s = singlet, d = doublet, t = triplet, p = pentet, and m = multiplet. Thin-layer chromatography (TLC) was performed using Whatman silica gel 60 Å plates with florescent indicator and visualized using a UV lamp (254 nm) or KMnO4 stain. Flash chromatography was performed on Grace with GraceResolv Normal Phase disposable silica columns. High-performance liquid chromatography (HPLC) was performed on a Gilson 322 HPLC pump with a Gilson UV/VIS-155 detector and a Phenomenex Gemini C_18_ column (10 μm, 250 mm × 10 mm). Liquid chromatography-electrospray ionization mass spectroscopy (LC-MS/ESI-MS) were acquired on an Agilent LC/MSD-SL with a 1100 HPLC and G1956B mass spectrometer with a Phenomenex Gemini 5 μm C18 110 Å 50 × 3 mm column. High-resolution mass spectra (HRMS-ESI) were acquired by the Mass Spectrometry Laboratory at the University of Michigan on an Agilent Q-TOF HPLC-MS. Purity for molecules was determined by HPLC (as above). In general, molecules were >95% pure, except as stated for molecules 17 and 20. Molecules used in key assays were all >95% pure.

### Reaction scheme for synthesis.

General procedure for the synthesis of compounds (excluding 15, 25, and 27): appropriate amine (1.0 eq), alkyl isothiocyanate (1.1 eq to 1.2 eq), triethylamine (2.0 eq), and anhydrous dimethylformamide (DMF) were stirred. The reaction mixture was heated from 90°C to 100°C and monitored by TLC and LC-MS. Saturated sodium chloride solution and ethyl acetate were added to the reaction mixture. The aqueous layer was extracted with ethyl acetate. The combined organic layer was dried over anhydrous sodium sulfate and concentrated *in vacuo*. The crude reaction mixture was purified via column chromatography and recrystallization.

**(i) IDR-0167255/TPN-0002034 (compound 1).** Compound 1 was 1-(benzo[d]thiazol-2-yl)-3-butylthiourea. Triethylamine (0.75 mL, 5.38 mmol) and 2-aminobenzothiazole (0.3998 g, 2.6618 mmol) were added to a stirred solution of butyl isothiocyanate (0.39 mL, 3.23 mmol) and DMF (5.4 mL). The reaction mixture was heated to 90°C and stirred for 18 h. The reaction mixture was transferred to the separatory funnel with EtOAc and saturated NaCl (aq) solution. The aqueous layer was extracted three times with EtOAc. The combined organic layer was dried over anhydrous Na_2_SO_4_, and the solvent was removed *in vacuo*. The product was purified by flash column chromatography (SiO_2_; hexanes:EtOAc 95:5 to 0:1) followed by recrystallization in MeOH to yield 1 as colorless crystals (0.0167 g, 2.4%). ^1^H NMR (300 MHz, DMSO-d6) δ 0.97 (t, *J* = 7.3 Hz, 3H), 1.42 (m, 2H), 1.53 to 1.74 (m, 2H), 3.62 (m, 2H), 7.21 to 7.53 (m, 2H), 7.72 (s, 1H), 7.96 (s, 1H), 10.16 (s, 1H), 11.80 (s, 1H); and HRMS-ESI (*m/z*): [M + H]^+^ calculated for C12H15N3S2, 266.0790, found 266.0784. Purity was 99%.

**(ii) IDR-0532302/TPN-0002029 (compound 11).** Compound 11 was 1-(benzo[d]imidazol-2-yl)-3-butylthiourea. Triethylamine (0.56 mL, 4 mmol) and butyl isothiocyanate (0.26 mL, 2.2 mmol) were added to a stirred solution of the 2-aminobenzimidazole (0.266 g, 2 mmol) and DMF (10 mL). The reaction mixture was heated to 100°C and stirred for 15 h. The reaction mixture was transferred to the separatory funnel with EtOAc and saturated NaCl (aq) solution. The aqueous layer was extracted three times with EtOAc. The combined organic layer was dried over anhydrous Na_2_SO_4_, and the solvent was removed *in vacuo*. The product was purified by recrystallization in ethanol to yield 11 as white crystals (0.042 g, 8.5%). ^1^H NMR (300 MHz, DMSO-d6) δ 0.97 (t, *J* = 7.3 Hz, 3H), 1.35 to 1.47 (m, 2H), 1.58 to 1.69 (m, 2H), 3.62 to 3.67 (m, 2H), 7.07 to 7.13 (m, 2H), 7.42 to 7.47 (m, 2H), 10.97 (s, 1H), 11.14 (s, 1H), 11.26 (s, 1H); HRMS-ESI (*m/z*): [M + H]^+^ calculated for C12H16N4S, 249.1168, found 249.1170. Purity was >95%.

**(iii) IDR-0532358/TPN-0002027 (compound 12).** Compound 12 was 1-(benzo[d]oxazol-2-yl)-3-butylthiourea. Triethylamine (0.56 mL, 4 mmol) and butyl isothiocyanate (0.26 mL, 2.2 mmol) were added to a stirred solution of the 2-aminobenzoxazole (0.268 g, 2 mmol) and DMF (10 mL). The reaction mixture was heated to 100°C and stirred for 15 h. The reaction mixture was transferred to the separatory funnel with EtOAc and saturated NaCl (aq) solution. The aqueous layer was extracted three times with EtOAc. The combined organic layer was dried over anhydrous Na_2_SO_4_, and the solvent was removed *in vacuo*. The product was purified by recrystallization in ethanol to yield 12 as white crystals (0.044 g, 9%). ^1^H NMR (300 MHz, DMSO-d6) δ 0.94 (t, *J* = 7.3 Hz, 3H), 1.33 to 1.46 (m, 2H), 1.60 to 1.70 (m, 2H), 3.65 to 3.70 (m, 2H), 7.25 to 7.35 (m, 2H), 7.56 to 7.63 (m, 2H), 10.51 (s, 1H), 12.28 (s, 1H); HRMS-ESI (*m/z*): [M + H]^+^ calculated for C12H15N3OS, 250.1020, found 250.1012. Purity was 99%.

**(iv) IDR-0532303/TPN-0002028 (compound 13).** Compound 13 was 1-butyl-3-(4-chlorobenzo[d]thiazol-2-yl)thiourea. Triethylamine (0.28 mL, 2 mmol) and butyl isothiocyanate (0.13 mL, 1.1 mmol) were added to a stirred solution of the 2-amino-4-chlorobenzthiazole (0.184 g, 1 mmol) and DMF (5 mL). The reaction mixture was heated to 100°C and stirred for 15 h. The reaction mixture was transferred to the separatory funnel with EtOAc and saturated NaCl (aq) solution. The aqueous layer was extracted three times with EtOAc. The combined organic layer was dried over anhydrous Na_2_SO_4_, and the solvent was removed *in vacuo*. The product was purified by recrystallization in ethanol to yield 13 as white crystals (0.042 g, 14%). ^1^H NMR (300 MHz, DMSO-d6) δ 0.96 (t, *J* = 7.3 Hz, 3H), 1.36 to 1.48 (m, 2H), 1.56 to 1.66 (m, 2H), 3.53 to 3.60 (m, 2H), 7.24 to 7.31 (m, 1H), 7.50 to 7.54 (m, 1H), 7.91 to 7.94 (m, 1H), 9.63 (s, 1H), 12.14 (s, 1H); HRMS-ESI (*m/z*): [M + H]^+^ calculated for C12H14ClN3S2, 300.0400, found 300.0393. Purity was 99%.

**(v) IDR-0532394/TPN-0002022 (compound 14).** Compound 14 was 1-butyl-3-(thiazol-2-yl)thiourea. Triethylamine (0.56 mL, 4 mmol) and butyl isothiocyanate (0.26 mL, 2.2 mmol) were added to a stirred solution of the 2-aminothiazole (0.200 g, 2 mmol) and DMF (10 mL). The reaction mixture was heated to 100°C and stirred for 15 h. The reaction mixture was transferred to the separatory funnel with EtOAc and saturated NaCl (aq) solution. The aqueous layer was extracted three times with EtOAc. The combined organic layer was dried over anhydrous Na_2_SO_4_, and the solvent was removed *in vacuo*. The product was purified by flash column chromatography (SiO_2_; hexanes:EtOAc 1:0 to 0:1) to yield 14 as white powder (0.086 g, 20%). ^1^H NMR (300 MHz, DMSO-d6) δ 0.91 (t, *J* = 7.3 Hz, 3H), 1.28 to 1.40 (m, 2H), 1.51 to 1.60 (m, 2H), 3.49 to 3.55 (m, 2H), 7.09 to 7.12 (m, 1H), 7.39 to 7.43 (m, 1H), 9.66 (s, 1H), 11.52 (s, 1H); HRMS-ESI (*m/z*): [M + H]^+^ calculated for C8H13N3S2, 216.0630, found 216.0623. Purity was 99%.

**FIG 5:**
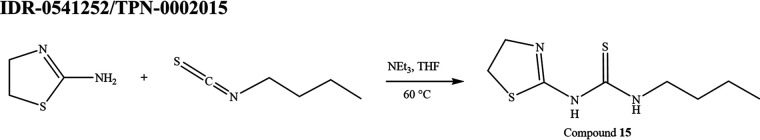
Synthesis scheme for compound 15.

**(vi) IDR-0541252/TPN-0002015 (compound 15).** Compound 15 was 1-butyl-3-(4,5-dihydrothiazol-2-yl)thiourea (see Fig. 5). Triethylamine (0.20 mL, 1.43 mmol) and 2-amino-2-thiazoline (0.1923 g, 1.387 mmol) were added to a stirred solution of butyl isothiocyanate (0.20 mL, 1.38 mmol) and tetrahydrofuran (2.8 mL). The reaction mixture was heated to 60°C and stirred overnight. The reaction mixture was extracted three times with CH2Cl2 and once with saturated NaCl (aq). The organic layers were combined and dried over anhydrous Na_2_SO_4_, and the solvent was removed *in vacuo*. The product was purified by flash column chromatography (SiO_2_; hexanes:EtOAc 4:1 and CH_2_Cl_2_:MeOH 95:5) to yield 15 as white solids (0.0221 g, 7.4%). ^1^H NMR (300 MHz, methylene chloride-d2) δ 0.92 (t, *J* = 7.4 Hz, 3H), 1.19 to 1.45 (m, 2H), 1.45 to 1.67 (m, 2H), 3.12 to 3.43 (m, 2H), 3.54 (d, *J* = 6.6 Hz, 2H), 3.84 (overlapping t, 2H), 4.02 (s), 8.20 (s, 1H), 11.02 (s, 1H); HRMS-ESI (*m/z*): [M + H]^+^ calculated for C8H15N3S2, 218.0790, found 218.0786. Purity was 95%.

**(vii) IDR-0566795/TPN-0002011 (compound 16).** Compound 16 was 1-butyl-3-(4-methylthiazol-2-yl)thiourea. Triethylamine (0.73 mL, 5.24 mmol) and 2-amino-4-methyl-thiazole (0.3087 g, 2.7039 mmol) were added to a stirred solution of butyl isothiocyanate (0.40 mL, 3.32 mmol) and DMF (5.3 mL). The reaction mixture was heated to 90°C and stirred for 5 h. The reaction mixture was transferred to the separatory funnel with EtOAc and H_2_O. The aqueous layer was extracted three times with EtOAc. The combined organic layer was extracted once with saturated NaCl (aq) and dried over anhydrous Na_2_SO_4_, and the solvent was removed *in vacuo*. The product was purified by flash column chromatography (SiO_2_; hexanes:EtOAc 9:1 to 0:1) followed by recrystallization in EtOH to yield 16 as colorless crystals (0.0268 g, 4.5%). ^1^H NMR (300 MHz, DMSO-d6) δ 0.95 (t, *J* = 7.3 Hz, 3H), 1.38 (h, 7.4 Hz, 2H), 1.59 (p, *J* = 7.1 Hz, 2H), 2.26 (s, 3H), 3.56 (q, *J* = 6.5 Hz, 2H), 6.70 (s, 1H), 9.98 (s, 1H), 11.49 (s, 1H); HRMS-ESI (*m/z*): [M + H]^+^ calculated for C9H15N3S2, 230.0790, found 230.0784. Purity was 99%.

**(viii) IDR-0566794/TPN-0002012 (compound 17).** Compound 17 was 1-butyl-3-(4,5-dimethylthiazol-2-yl)thiourea. Triethylamine (0.65 mL, 4.66 mmol) and 4,5-dimethyl-1,3-thiazol-2-amine (0.3060 g, 2.3869 mmol) were added to a stirred solution of butyl isothiocyanate (0.35 mL, 2.90 mmol) and DMF (4.7 mL). The reaction mixture was heated to 90°C and stirred for 4 h. The reaction mixture was transferred to the separatory funnel with EtOAc and H_2_O. The aqueous layer was extracted three times with EtOAc. The combined organic layer was extracted once with saturated NaCl (aq) and dried over anhydrous Na_2_SO_4_, and the solvent was removed *in vacuo*. The product was purified by flash column chromatography (SiO_2_; hexanes:EtOAc 9:1 to 0:1) followed by recrystallization in EtOH to yield 17 as colorless crystals (0.0255 g, 4.5%). ^1^H NMR (300 MHz, DMSO-d6) δ 0.94 (t, *J* = 7.3 Hz, 3H), 1.38 (h, *J* = 7.2 Hz, 2H), 1.58 (p, *J* = 7.2 Hz, 2H), 2.15 (s, 3H), 2.22 (s, 3H), 3.54 (m, 2H), 10.00 (s, 1H), 11.35 (s, 1H); HRMS-ESI (*m/z*): [M + H]^+^ calculated for C10H17N3S2, 244.0940, found 244.0941. Purity was 93%.

**(ix) IDR-0563572/TPN-0002013 (compound 18).** Compound 18 was 1-butyl-3-(4-methyl-1H-imidazol-2-yl)thiourea. Triethylamine (0.72 mL, 5.2 mmol) and 5-methyl-1H-imidazol-2-ylamine (0.2504 g, 2.578 mmol) were added to a stirred solution of butyl isothiocyanate (0.37 mL, 3.1 mmol) and DMF (5.0 mL). The reaction mixture was heated to 90°C and stirred for 18 h. The reaction mixture was transferred to the separatory funnel with EtOAc and saturated NaCl (aq) solution. The aqueous layer was extracted three times with EtOAc. The combined organic layer was dried over anhydrous Na_2_SO_4_, and the solvent was removed *in vacuo*. The product was purified by flash column chromatography (SiO_2_; hexanes:EtOAc 95:5 to 0:1) followed by HPLC (water:MeCN 0 to 100) to yield 18 as a yellow oil (0.0184 g, 3.3%). ^1^H NMR (300 MHz, DMSO-d6) δ 0.94 (t, *J* = 7.3 Hz, 3H), 1.39 (h, 7.1 Hz, 2H), 1.58 (p, *J* = 7.1 Hz, 2H), 2.10 (s, 3H), 3.58 (q, *J* = 6.7 Hz, 2H), 6.49 (s, 1H), 10.05 to 11.20 (m, 3H); LC-MS-ESI (*m/z*): [M + H]^+^ calculated for C12H14N2OS2, 267.0630, found 267.0627. Purity was 99%.

**(x) IDR-0532542/TPN-0002016 (compound 19).** Compound 19 was 1-butyl-3-(1-methyl-1H-pyrazol-5-yl)thiourea. Triethylamine (0.28 mL, 2 mmol) and butyl isothiocyanate (0.13 mL, 1.1 mmol) were added to a stirred solution of the 5-amino-1-methylpyrazole (0.097 g, 1 mmol) and DMF (5 mL). The reaction mixture was heated to 100°C and stirred for 8 h. The reaction mixture was transferred to the separatory funnel with EtOAc and saturated NaCl (aq) solution. The aqueous layer was extracted three times with EtOAc. The combined organic layer was dried over anhydrous Na_2_SO_4_, and the solvent was removed *in vacuo*. The product was purified by flash column chromatography (SiO_2_; hexanes:EtOAc 1:0 to 0:1) to yield 19 as white powder (0.046 g, 22%). ^1^H NMR (300 MHz, chloroform-d) δ 0.93 (t, *J* = 7.3 Hz, 3H), 1.26 to 1.38 (m, 2H), 1.50 to 1.60 (m, 2H), 3.58 to 3.64 (m, 2H), 3.79 (s, 3H), 5.80 to 6.10 (m, 1H), 6.16 to 6.17 (m, 1H), 7.52 (s, 1H), 7.96 (s, 1H); HRMS-ESI (*m/z*): [M + H]^+^ calculated for C9H16N4S, 213.1180, found 213.1171. Purity was 99%.

**(xi) IDR-0571506/TPN-0002004 (compound 20).** Compound 20 was 1-butyl-3-(4-methylpyrimidin-2-yl)thiourea. Triethylamine (1.0 mL, 7.2 mmol) and 2-amino-4-methylpyrimidine (0.4004 g, 3.6690 mmol) were added to a stirred solution of butyl isothiocyanate (0.52 mL, 4.31 mmol) and DMF (7.2 mL). The reaction mixture was heated to 90°C and stirred for 19 h. The reaction mixture was transferred to the separatory funnel with EtOAc and saturated NaCl (aq) solution. The aqueous layer was extracted three times with EtOAc. The combined organic layer was dried over anhydrous Na_2_SO_4_, and the solvent was removed *in vacuo*. The product was purified by flash column chromatography (SiO_2_; hexanes:EtOAc 95:5 to 0:1) followed by recrystallization in MeOH to yield 20 as white solids (0.0165 g, 2.0%). ^1^H NMR (300 MHz, DMSO-d6) δ 0.96 (t, *J* = 7.3 Hz, 3H), 1.42 (h, *J* = 7.4 Hz, 2H), 1.65 (p, *J* = 7.0 Hz, 2H), 3.64 (q, *J* = 6.9, 2H), 7.08 (d, *J* = 5.1 Hz, 1H), 8.53 (d, *J* = 5.1 Hz, 1H), 10.49 (s, 1H), 11.40 (s, 1H); HRMS-ESI (*m/z*): [M + H]^+^ calculated for C10H16N4S, 225.1180, found 225.1169. Purity was 90%.

**(xii) IDR-0571504/TPN-0002005 (compound 21).** Compound 21 was 1-butyl-3-(4-methyloxazol-2-yl)thiourea. Triethylamine (1.15 mL, 8.25 mmol) and 2-amino-4-methyloxazole (0.36 mL, 4.29 mmol) were added to a stirred solution of butyl isothiocyanate (0.60 mL, 4.97 mmol) and DMF (8.2 mL). The reaction mixture was heated to 90°C and stirred for 19 h. The reaction mixture was transferred to the separatory funnel with EtOAc and saturated NaCl (aq) solution. The aqueous layer was extracted three times with EtOAc. The combined organic layer was dried over anhydrous Na_2_SO_4_, and the solvent was removed *in vacuo*. The product was purified by flash column chromatography (SiO_2_; hexanes:EtOAc 95:5 to 0:1) to yield 21 as light yellow solids (0.0465 g, 5.1%). ^1^H NMR (300 MHz, DMSO-d6) δ 0.95 (t, *J* = 7.3 Hz, 3H), 1.39 (h, *J* = 7.4 Hz, 2H), 1.61 (p, *J* = 7.2 Hz, 2H), 2.08 (s, 3H), 3.62 (q, *J* = 6.6 Hz, 2H), 7.53 (s, 1H), 10.34 (s, *J* = 5.6 Hz, 1H), 11.80 (s, 1H); HRMS-ESI (*m/z*): [M + H]^+^ calculated for C9H15N3OS, 214.1020, found 214.1011. Purity was 99%.

**(xiii) IDR-0578471/TPN-0002000 (compound 22).** Compound 22 was 1-butyl-3-(5-methyloxazol-2-yl)thiourea. Triethylamine (1.1 mL, 7.9 mmol) and 5-methyl-1,3-oxazole-2-amine (0.3930 g, 4.006 mmol) were added to a stirred solution of butyl isothiocyanate (0.60 mL, 5.0 mmol) and DMF (8.0 mL). The reaction mixture was heated to 90°C and stirred for 24 h. The reaction mixture was transferred to the separatory funnel with EtOAc and saturated NaCl (aq) solution. The aqueous layer was extracted three times with EtOAc. The combined organic layer was dried over anhydrous Na_2_SO_4_, and the solvent was removed *in vacuo*. The product was purified by flash column chromatography (SiO_2_; hexanes:EtOAc 95:5 to 0:1 and 95:5 CH2Cl2:MeOH) followed by recrystallization in MeOH to yield 22 as beige-colored solids (0.0851 g, 9.8%). ^1^H NMR (300 MHz, DMSO-d6) δ 0.94 (t, *J* = 7.3 Hz, 3H), 1.37 (h, *J* = 7.3 Hz, 2H), 1.60 (p, *J* = 7.1 Hz, 2H), 2.26 (s, 3H), 3.61 (q, *J* = 6.6 Hz, 2H), 6.78 (s, 1H), 10.30 (s, 1H), 11.76 (s, 1H); HRMS-ESI (*m/z*): [M + H]^+^ calculated for C9H15N3OS, 214.1020, found 214.1011. Purity was 99%.

**(xiv) IDR-0578334/TPN-0002001 (compound 23).** Compound 23 was 1-butyl-3-(4-phenyloxazol-2-yl)thiourea. Triethylamine (0.80 mL, 5.74 mmol) and 4-phenyl-1,3-oxazol-2-amine (0.2475 g, 1.545 mmol) were added to a stirred solution of butyl isothiocyanate (0.42 mL, 3.48 mmol) and DMF (5.6 mL). The reaction mixture was heated to 90°C and stirred for 28 h. The reaction mixture was transferred to the separatory funnel with EtOAc and saturated NaCl (aq) solution. The aqueous layer was extracted three times with EtOAc. The combined organic layer was dried over anhydrous Na_2_SO_4_, and the solvent was removed *in vacuo*. The product was purified by flash column chromatography (SiO_2_; hexanes:EtOAc 95:5 to 0:1 and CH2Cl2:MeOH 9:1 to 0:1) followed by HPLC (water:MeCN 0 to 100) to yield 23 as white solids (0.0042 g, 1%). ^1^H NMR (300 MHz, DMSO-d6) δ 1.00 (t, *J* = 7.3 Hz, 3H), 1.48 (h, *J* = 7.9 Hz, 2H), 1.69 (p, *J* = 7.2, 6.8 Hz, 2H), 3.68 (q, *J* = 6.4 Hz, 2H), 7.43 (m, 3H), 7.77 (d, *J* = 7.6 Hz, 2H), 8.36 (s, 1H), 10.46 (s, 1H), 11.99 (s, 1H); HRMS-ESI (*m/z*): [M + H]^+^ calculated for C14H17N3OS, 276.1170, found 276.1170. Purity was 99%.

**(xv) IDR-0532359/TPN-0002026 (compound 24).** Compouned 24 was 1-(benzo[d]thiazol-2-yl)-3-(3-methoxypropyl)thiourea. Triethylamine (0.28 mL, 2 mmol) and 3-methoxypropyl isothiocyanate (0.14 mL, 1.1 mmol) were added to a stirred solution of the 2-aminobenzothiazole (0.150 g, 1 mmol) and DMF (5 mL). The reaction mixture was heated to 100°C and stirred for 15 h. The reaction mixture was transferred to the separatory funnel with EtOAc and saturated NaCl (aq) solution. The aqueous layer was extracted three times with EtOAc. The combined organic layer was dried over anhydrous Na_2_SO_4_, and the solvent was removed *in vacuo*. The product was purified by recrystallization in ethanol to yield 24 as white crystals (0.029 g, 10%). ^1^H NMR (300 MHz, DMSO-d6) δ 1.82 to 1.90 (m, 2H), 3.27 (s, 3H), 3.41 to 3.46 (m, 2H), 3.61 to 3.70 (m, 2H), 7.26 to 7.93 (m, 4H), 10.16 (s, 1H), 10.81 (s, 1H); HRMS-ESI (*m/z*): [M + H]^+^ calculated for C12H15N3OS2, 282.0740, found 282.0734. Purity was >95%.

**FIG 6:**
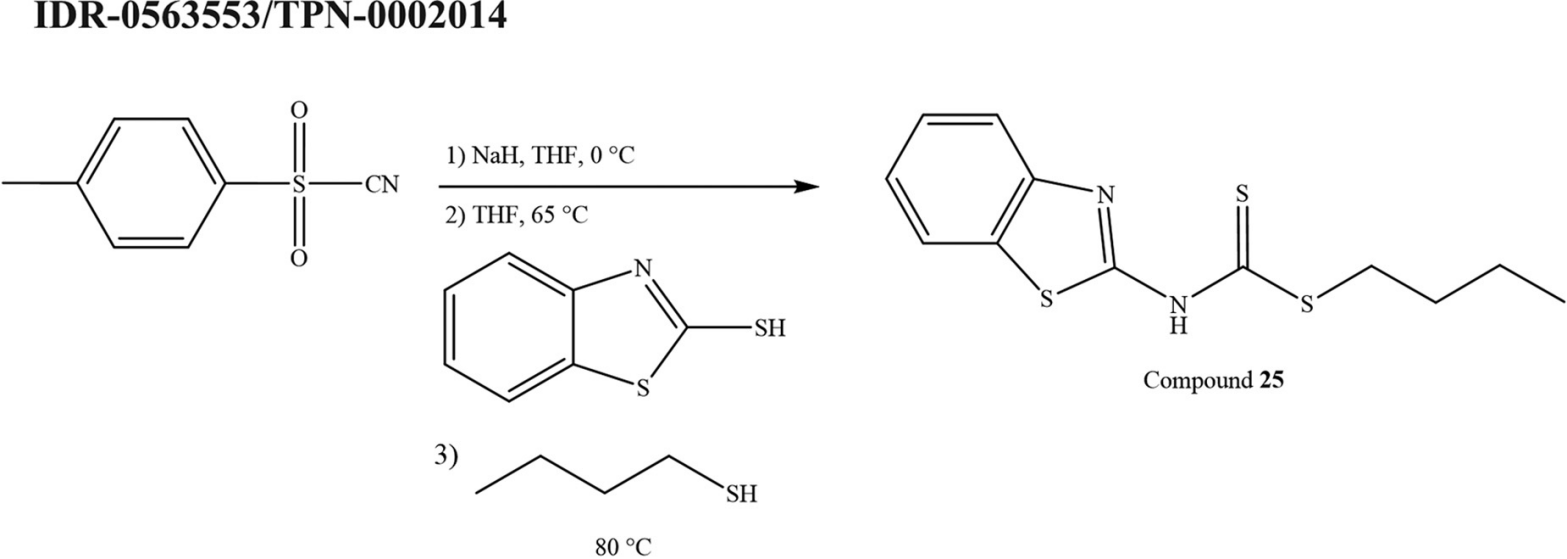
Synthesis scheme for compound 25.

**(xvi) IDR-0563553/TPN-0002014 (compounds 25a and 25b).** Compound 25a was 2-thiocyanatobenzo[d]thiazole (see Fig. 6). Sodium hydride (0.4482 g, 18.68 mmol) was added to a solution of p-toluenesulfonyl cyanide (1.64 g, 9.05 mmol) in tetrahydrofuran (THF) (6.0 mL) while stirring in an ice-water bath. A solution of 2-mercaptobenzothiazole (1.0112 g, 6.0460 mmol) in THF (6.0 mL) was added to the reaction mixture. The reaction mixture was heated to 65°C and stirred for 2.5 h. The reaction mixture was cooled in an ice-water bath and quenched with water. The aqueous layer was extracted three times with EtOAc. The combined organic layers were dried over anhydrous Na_2_SO_4_ and the solvent was removed *in vacuo* to yield 1.65 g of solid.

Compound 25b was butyl benzo[d]thiazol-2-ylcarbamodithioate. A solution of compound 25a (0.4013 g, 2.0873 mmol) and 1-butanethiol (3.0 mL, 28 mmol) was heated to 80°C while stirring. The reaction mixture was stirred for 3 h. The reaction mixture was extracted three times with CH2Cl2 and once with saturated NaCl (aq). The organic layers were combined and dried over anhydrous Na_2_SO_4_, and the solvent was removed *in vacuo*. The product was purified by flash column chromatography (SiO_2_; hexanes:EtOAc 4:1) to yield 25 as yellow solids (0.0269 g, 4.6%). ^1^H NMR (300 MHz, chloroform-d) δ 0.97 (t, *J* = 7.3 Hz, 3H), 1.47 (h, *J* = 14.5, 7.4 Hz, 2H), 1.74 (p, *J* = 15.2, 7.0 Hz, 2H), 3.33 (t, *J* = 7.5 Hz, 2H), 7.40 (t, *J* = 7.9 Hz, 1H), 7.51 (t, *J* = 7.7 Hz, 1H), 7.77 (dd, *J* = 21.2, 8.0 Hz, 2H); HRMS-ESI (*m/z*): [M + H]^+^ calculated for C12H14N2S3, 283.0400, found 283.0397. Purity was 95%.

**(xvii) IDR-0532360/TPN-0002025 (compound 26).** Compound 26 was 1-(benzo[d]thiazol-2-yl)-3-phenethylthiourea. Triethylamine (0.28 mL, 2 mmol) and 2-phenylethyl isothiocyanate (0.16 mL, 1.1 mmol) were added to a stirred solution of the 2-aminobenzothiazole (0.150 g, 1 mmol) and DMF (5 mL). The reaction mixture was heated to 100°C and stirred for 15 h. The reaction mixture was transferred to the separatory funnel with EtOAc and saturated NaCl (aq) solution. The aqueous layer was extracted three times with EtOAc. The combined organic layer was dried over anhydrous Na_2_SO_4_, and the solvent was removed *in vacuo*. The product was purified by recrystallization in ethanol to yield 29 as white crystals (0.035 g, 11%). ^1^H NMR (300 MHz, DMSO-d6) δ 2.94 to 2.99 (m, 2H), 3.84 to 3.86 (m, 2H), 7.24 to 7.44 (m, 7H), 7.57 to 7.60 (m, 1H), 7.89 to 7.92 (m, 1H), 10.27 (s, 1H), 11.86 (s, 1H); HRMS-ESI (*m/z*): [M + H]^+^ calculated for C16H15N3S2, 314.0790, found 314.0783. Purity was 99%.

**FIG 7:**
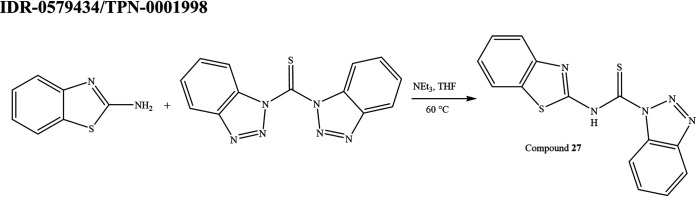
Synthesis scheme for compound 27.

**(xviii) IDR-0579434/TPN-0001998 (compound 27).** Compound 27 was 3-(benzo[d]thiazol-2-yl)-1-butyl-1-methylthiourea (see Fig. 7). Bis(1-benzotriazolyl)methanethione (0.5051 g, 1.802 mmol) was added to a stirred solution of 2-aminobenzothiazole (0.2199 g, 1.464 mmol) and THF (3.0 mL). The reaction mixture was stirred at ambient temperature for 21 h. Triethylamine (0.41 mL, 2.9 mmol), and *N*-methylbutylamine (0.21 mL, 1.8 mmol) were added to the reaction mixture and the reaction was left to stir for another 32 h at ambient temperature, and at 50°C for 42 h. The reaction was monitored by LC-MS and TLC. The reaction mixture was transferred to the separatory funnel with EtOAc and water. The aqueous layer was extracted three times with EtOAc. The combined organic layer was dried over anhydrous Na_2_SO_4_, and the solvent was removed *in vacuo*. The product was purified by flash column chromatography (SiO_2_; hexanes:EtOAc 95:5 to 0:1) followed by HPLC (water:MeCN 0 to 100) to yield 27 as a white solid (0.1149 g, 28.1%). ^1^H NMR (300 MHz, DMSO-d6) δ 0.96 (t, *J* = 7.4 Hz, 3H), 1.34 (m, 2H), 1.61 (p, *J* = 9.0 Hz, 2H), 3.33 (s, 3H), 3.89 (t, *J* = 7.5 Hz, 2H), 7.13 to 7.48 (m, 3H), 7.76 (d, *J* = 6.3 Hz, 1H), 12.71 (s, 1H); LC-MS-ESI (*m/z*): [M + H]^+^ calculated for C13H18N3S2, 280.1, found 280.0. Purity was 99%.

**(xix) IDR-0532392/TPN-0002024 (compound 28).** Compound 28 was 1-(benzo[d]thiazol-2-yl)-3-isobutylthiourea. Triethylamine (0.56 mL, 4 mmol) and isobutyl isothiocyanate (0.28 mL, 2.2 mmol) were added to a stirred solution of the 2-aminobenzothiazole (0.3 g, 2 mmol) and DMF (10 mL). The reaction mixture was heated to 100°C and stirred for 15 h. The reaction mixture was transferred to the separatory funnel with EtOAc and saturated NaCl (aq) solution. The aqueous layer was extracted three times with EtOAc. The combined organic layer was dried over anhydrous Na_2_SO_4_, and the solvent was removed *in vacuo*. The product was purified by recrystallization in ethanol to yield 28 as white crystals (0.052 g, 10%). ^1^H NMR (300 MHz, DMSO-d6) δ 0.96 to 0.98 (d, *J* = 6.6 Hz, 6×H), 1.94 to 2.03 (m, 1H), 3.20 to 3.44 (m, 2H), 7.26 to 7.41 (m, 2H), 7.65 to 7.91 (m, 2H), 10.25 (s, 1H), 11.81 (s, 1H); HRMS-ESI (*m/z*): [M + H]^+^ calculated for C12H15N3S2, 266.0790, found 266.0796. Purity was >95%.

**(xx) IDR-0578333/TPN-0002002 (compound 29).** Compound 29 was 1-isobutyl-3-(4-methyloxazol-2-yl)thiourea. Triethylamine (1.4 mL, 10 mmol) and 2-amino-4-methyloxazole (0.43 mL, 5.1 mmol) were added to a stirred solution of isobutyl isothiocyanate (0.75 mL, 6.1 mmol) and DMF (10.0 mL). The reaction mixture was heated to 90°C and stirred for 23 h. The reaction mixture was transferred to the separatory funnel with EtOAc and saturated NaCl (aq) solution. The aqueous layer was extracted three times with EtOAc. The combined organic layer was dried over anhydrous Na_2_SO_4_, and the solvent was removed *in vacuo*. The product was purified by flash column chromatography (SiO_2_; hexanes:EtOAc 95:5 to 0:1 and CH2Cl2:MeOH 95:5 to 0:1) followed by HPLC (water:MeCN 0 to 100) to yield 29 as white solids (0.0132 g, 1.2%). ^1^H NMR (300 MHz, DMSO-d6) δ 0.98 (d, *J* = 6.7 Hz, 6 × H),1.87 to 2.15 (m, 4H), 3.48 (t, *J* = 6.0 Hz, 2H), 7.54 (s, 1H), 10.46 (s, 1H), 11.83 (s, 1H); HRMS-ESI (*m/z*): [M + H]^+^ calculated for C9H15N3OS, 214.1020, found 214.1007. Purity was 99%.

**(xxi) IDR-0571381/TPN-0002010 (compound 30).** Compund 30 was 1-(benzo[d]thiazol-5-yl)-3-butylthiourea. Triethylamine (0.65 mL, 4.66 mmol) and 5-aminobenzothiazole (0.3494 g, 2.3262 mmol) were added to a stirred solution of butyl isothiocyanate (0.34 mL, 2.82 mmol) and DMF (4.5 mL). The reaction mixture was heated to 90°C and stirred for 6.5 h. The reaction mixture was transferred to the separatory funnel with EtOAc and H_2_O. The aqueous layer was extracted three times with EtOAc. The combined organic layer was extracted once with saturated NaCl (aq), dried over anhydrous Na_2_SO_4_, and the solvent was removed *in vacuo*. The product was purified by flash column chromatography (SiO_2_; CH_2_Cl_2_:MeOH 1:0 to 9:1) followed by recrystallization in acetone to yield 30 as white solids (0.0479 g, 7.7%). ^1^H NMR (300 MHz, DMSO-d6) δ 0.95 (t, *J* = 7.3 Hz, 3H), 1.37 (h, *J* = 7.6 Hz, 2H), 1.57 (p, *J* = 7.4 Hz, 2H), 3.51 (q, *J* = 6.8 Hz, 2H), 7.48 (d, *J* = 8.9 Hz, 1H), 7.90 (s, 1H), 8.11 (d, *J* = 8.6 Hz, 1H), 8.23 (s, 1H), 9.42 (s, 1H), 9.67 (s, 1H); HRMS-ESI (*m/z*): [M + H]^+^ calculated for C12H15N3S2, 266.0790, found 266.0786. Purity was 99%.

**(xxii) IDR-0532393/TPN-0002023 (compound 31).** Compound 31 was 1-(4-chlorobenzo[d]thiazol-2-yl)-3-propyl thiourea. Triethylamine (0.28 mL, 2 mmol) and propyl isothiocyanate (0.11 mL, 1.1 mmol) were added to a stirred solution of the 2-amino-4-chlorobenzthiazole (0.184 g, 1 mmol) and DMF (5 mL). The reaction mixture was heated to 100°C and stirred for 15 h. The reaction mixture was transferred to the separatory funnel with EtOAc and saturated NaCl (aq) solution. The aqueous layer was extracted three times with EtOAc. The combined organic layer was dried over anhydrous Na_2_SO_4_, and the solvent was removed *in vacuo*. The product was purified by recrystallization in ethanol to yield 31 as white crystals (0.030 g, 10.5%). ^1^H NMR (300 MHz, DMSO-d6) δ 0.97 (t, *J* = 7.3 Hz, 3H), 1.57 to 1.69 (m, 2H), 3.50 to 3.56 (m, 2H), 7.25 to 7.30 (m, 1H), 7.49 to 7.53 (m, 1H), 7.91 to 7.94 (m, 1H), 9.56 (s, 1H), 12.13 (s, 1H); HRMS-ESI (*m/z*): [M + H]^+^ calculated for C11H12ClN3S2, 286.0240, found 286.0241. Purity was 98%.

### (xxiii) IDR-0571501/TPN-0002008 (compound 32).

Compound 32 was 1-butyl-3-(5-chlorobenzo[d]oxazol-2-yl) thiourea. Triethylamine (0.33 mL, 2.37 mmol) and 2-amino-5-chlorobenzoxazole (0.3998 g, 2.3716 mmol) were added to a stirred solution of butyl isothiocyanate (0.30 mL, 2.49 mmol) and DMF (4.6 mL). The reaction mixture was heated to 90°C and stirred for 23 h. The reaction mixture was transferred to the separatory funnel with EtOAc and saturated NaCl (aq) solution. The aqueous layer was extracted three times with EtOAc. The combined organic layer was dried over anhydrous Na_2_SO_4_, and the solvent was removed *in vacuo*. The product was purified by flash column chromatography (SiO_2_; hexanes:EtOAc 95:5 to 0:1) followed by recrystallization in CHCl3 to yield 32 as white solids (0.0382 g, 5.7%). ^1^H NMR (300 MHz, DMSO-d6) δ 0.97 (t, *J* = 7.3 Hz, 3H), 1.43 (h, *J* = 7.9 Hz, 2H), 1.67 (p, *J* = 7.3 Hz, 2H), 3.69 (q, *J* = 6.7 Hz, 2H), 7.35 (d, *J* = 6.2 Hz, 1H), 7.69 (d, *J* = 8.3 Hz, 2H), 10.42 (s, 1H), 12.45 (s, 1H); HRMS-ESI (*m/z*): [M + H]^+^ calculated for C12H14ClN3OS, 284.0630, found 284.0622. Purity was 98%.

**(xxiv) IDR-0571502/TPN-0002007 (compound 33).** Compound 33 was 1-butyl-3-(6-chlorobenzo[d]oxazol-2-yl)thiourea. Triethylamine (0.33 mL, 2.37 mmol) and 6-chloro-1,3-benzoxazole-2-amine (0.4005 g, 2.3757 mmol) were added to a stirred solution of butyl isothiocyanate (0.30 mL, 2.49 mmol) and DMF (4.6 mL). The reaction mixture was heated to 90°C and stirred for 26 h. The reaction mixture was transferred to the separatory funnel with EtOAc and saturated NaCl (aq) solution. The aqueous layer was extracted three times with EtOAc. The combined organic layer was dried over anhydrous Na_2_SO_4_, and the solvent was removed *in vacuo*. The product was purified by flash column chromatography (SiO_2_; hexanes:EtOAc 95:5 to 0:1) to yield 33 as white solids (0.0439 g, 6.5%). ^1^H NMR (300 MHz, DMSO-d6) δ 0.97 (t, *J* = 7.3 Hz, 3H), 1.42 (h, *J* = 7.4 Hz, 2H), 1.67 (p, *J* = 7.3 Hz, 2H), 3.69 (q, *J* = 6.6 Hz, 2H), 7.41 (d, *J* = 8.5, 1H), 7.61 (d, *J* = 8.4 Hz, 1H), 7.88 (s, 1H), 10.43 (s, 1H), 12.42 (s, 1H); HRMS-ESI (*m/z*): [M + H]^+^ calculated for C12H14ClN3OS, 284.0630, found 284.0621. Purity was 99%.

**(xxv) IDR-0571503/TPN-0002006 (compound 34).** Compound 34 was 1-butyl-3-(7-chlorobenzo[d]oxazol-2-yl)thiourea. Triethylamine (0.33 mL, 2.37 mmol) and 7-chloro-1,3-benzoxazole-2-amine (0.4003 g, 2.3745 mmol) were added to a stirred solution of butyl isothiocyanate (0.30 mL, 2.49 mmol) and DMF (4.6 mL). The reaction mixture was heated to 90°C and stirred for 24 h. The reaction mixture was transferred to the separatory funnel with EtOAc and saturated NaCl (aq) solution. The aqueous layer was extracted three times with EtOAc. The combined organic layer was dried over anhydrous Na_2_SO_4_, and the solvent was removed *in vacuo*. The product was purified by flash column chromatography (SiO_2_; hexanes:EtOAc 95:5 to 0:1) to yield 34 as white solids (0.0529 g, 7.9%). ^1^H NMR (300 MHz, DMSO-d6) δ 0.97 (t, *J* = 7.3 Hz, 3H), 1.43 (h, *J* = 7.5 Hz, 2H), 1.68 (p, *J* = 7.3 Hz, 2H), 3.69 (q, *J* = 6.6 Hz, 2H), 7.31 to 7.46 (m, 2H), 7.58 (d, *J* = 7.2 Hz, 1H), 10.40 (s, 1H), 12.45 (s, 1H); HRMS-ESI (*m/z*): [M + H]^+^ calculated for C12H14ClN3OS, 284.0630, found 284.0621. Purity 98%.

**(xxvi) IDR-0576402/TPN-0002003 (compound 35).** Compound 35 was 1-butyl-3-(5-methoxybenzo[d]oxazol-2-yl)thiourea. Triethylamine (0.70 mL, 5.0 mmol) and 5-methoxybenzo[d]oxazol-2-amine (0.3995 g, 2.4336 mmol) were added to a stirred solution of butyl isothiocyanate (0.35 mL, 2.90 mmol) and DMF (5.0 mL). The reaction mixture was heated to 90°C and stirred for 39 h. The reaction mixture was transferred to the separatory funnel with EtOAc and saturated NaCl (aq) solution. The aqueous layer was extracted three times with EtOAc. The combined organic layer was dried over anhydrous Na_2_SO_4_, and the solvent was removed *in vacuo*. The product was purified by flash column chromatography (SiO_2_; hexanes:EtOAc 95:5 to 0:1) to yield 35 as white solids (0.0810 g, 11.9%). ^1^H NMR (300 MHz, DMSO-d6) δ 0.97 (t, *J* = 7.3 Hz, 3H), 1.42 (h, *J* = 6.7 Hz, 2H), 1.67 (p, 6.9 Hz, 2H), 3.58 to 3.73 (q, *J* = 6.7 Hz, 2H), 3.83 (s, 3H), 6.95 (d, *J* = 6.9 Hz, 1H), 7.33 (s, 1H), 7.49 (d, *J* = 8.7 Hz, 1H), 10.46 (s, 1H), 12.23 (s, 1H); HRMS-ESI (*m/z*): [M + H]^+^ calculated for C13H17N3O2S, 280.1120, found 280.1118. Purity was 99%.
